# Serine Metabolism Regulates YAP Activity Through USP7 in Colon Cancer

**DOI:** 10.3389/fcell.2021.639111

**Published:** 2021-05-12

**Authors:** Xiaoya Zhao, Jianfei Fu, Bin Hu, Lin Chen, Jing Wang, Jinyong Fang, Chenyang Ge, Haiping Lin, Kailing Pan, Liang Fu, Lude Wang, Jinlin Du, Wenxia Xu

**Affiliations:** ^1^Central Laboratory, Affiliated Jinhua Hospital, Zhejiang University School of Medicine, Jinhua, China; ^2^Department of Medical Oncology, Sir Run Run Shaw Hospital, Zhejiang University School of Medicine, Hangzhou, China; ^3^Department of Medical Oncology, Affiliated Jinhua Hospital, Zhejiang University School of Medicine, Jinhua, China; ^4^Department of Pathology, Affiliated Jinhua Hospital, Zhejiang University School of Medicine, Jinhua, China; ^5^Department of Science and Education, Jinhua Guangfu Oncology Hospital, Huancheng, Jinhua, China; ^6^Department of Colorectal Surgery, Affiliated Jinhua Hospital, Zhejiang University School of Medicine, Jinhua, China; ^7^Department of Nursing, Affiliated Jinhua Hospital, Zhejiang University School of Medicine, Jinhua, China

**Keywords:** colon cancer, serine metabolism, YAP, USP7, organoid

## Abstract

Metabolic reprogramming is a vital factor in the development of many types of cancer, including colon cancer. Serine metabolic reprogramming is a major feature of tumor metabolism. Yes-associated protein (YAP) participates in organ size control and tumorigenesis. However, the relationship between YAP and serine metabolism in colon cancer is unclear. In this study, RNA sequencing and metabolomics analyses indicated significant enrichment of the glycine, serine, and threonine metabolism pathways in serine starvation–resistant cells. Short-term serine deficiency inhibited YAP activation, whereas a prolonged response dephosphorylated YAP and promoted its activity. Mechanistically, USP7 increases YAP stability under increased serine conditions by regulating deubiquitination. Verteporfin (VP) effectively inhibited the proliferation of colon cancer cells and organoids and could even modulate serine metabolism by inhibiting USP7 expression. Clinically, YAP was significantly activated in colon tumor tissues and positively correlated with the expression of phosphoglycerate dehydrogenase (PHGDH) and USP7. Generally, our study uncovered the mechanism by which serine metabolism regulates YAP via USP7 and identified the crucial role of YAP in the regulation of cell proliferation and tumor growth; thus, VP may be a new treatment for colon cancer.

## Introduction

Colon cancer, a malignant tumor of the colon, is often associated with rectal cancer; therefore, it is often called colorectal cancer (CRC). At present, colorectal cancer is the fourth most deadly cancer in the world, responsible for almost 900,000 deaths annually (Dekker et al., 2019). Despite considerable advancements in the treatments of CRC in recent years, the outcome of CRC remains unfavorable. The global incidence of CRC is predicted to rise to 2.5 million new cases in 2035 (Arnold et al., 2017; Bray et al., 2018). Therefore, knowledge of the mechanism of the occurrence and development of colon cancer should provide a scientific basis to identify effective prevention and treatment strategies.

Nutrition plays both causal and protective roles in the development of colon cancer (Thanikachalam and Khan, 2019). For example, amino acids play a crucial role in the biosynthesis of nucleotides, proteins, lipids, antioxidants, and tricarboxylic acid (TCA) cycle intermediates, all of which support the requirements for exponential cell growth and proliferation. Limited exogenous amino acids such as serine, glycine, and aspartate—non-essential amino acids (NEAAs)—can be synthesized *de novo* (Gui et al., 2016; Muir et al., 2017; Sullivan and Vander Heiden, 2017, 2019; Garcia-Bermudez et al., 2018; Sullivan et al., 2019a, b). Serine availability is a critical factor in the efficiency of many cellular processes, such as nucleotide synthesis, folate metabolism, and macromolecule synthesis (Mattaini et al., 2016). Highly proliferative cells exhibit a strong demand for serine; this can be satisfied by enhancing either import from the extracellular environment or *de novo* synthesis from glucose (Locasale et al., 2011). Serine biosynthesis occurs via the serine synthesis pathway (SSP) through phosphoglycerate dehydrogenase (PHGDH), converting 3-phosphoglyceric acid into 3-phosphatedehydropyruvate, which is subsequently catalyzed by phosphoserine aminotransferase 1 (PSAT1) and phosphoserine phosphatase (PSPH) (Zhao et al., 2020a). Notably, enhancement of the SSP is major metabolic reprogramming and is important for oncogenic transformation in many cancers, including melanoma, triple-negative breast cancer, subcutaneous lung cancer, and brain metastases (Possemato et al., 2011; DeNicola et al., 2015; Sullivan et al., 2019b; Ngo et al., 2020). PHGDH, PSAT1, and PSPH were reported to be overexpressed in colon cancer. Moreover, PHGDH was positively correlated with the TNM stage as well as the tumor size and was an independent predictor of poor prognosis in patients with colon cancer (Yoon et al., 2015; Jia et al., 2016; Ngo et al., 2020). However, the regulatory mechanism behind the altered serine metabolism in colon cancer remains incompletely understood, and the relationship between serine metabolic/mechanistic dysregulation and tumor growth is not well characterized.

Yes-associated protein (YAP), a key effector in the Hippo signaling pathway, regulates the proliferation, survival, and self-renewal ability of multiple human cancers, particularly colon cancer (Wang et al., 2013; Zhu et al., 2016; Eibl and Rozengurt, 2019; Lee et al., 2019). The accumulation of nuclear YAP and its paralog, transcriptional co-activator with PDZ-binding (TAZ), jointly triggers TEAD-dependent transcription gene expression (CTGF, CDX2, Cyclin D1, etc.), which is responsible for tumorigenesis, drug resistance, metastasis, and immune suppression (Wang et al., 2017, 2018; Chen et al., 2019). Recent findings have shown that YAP is inextricably related to components of tumor metabolism, such as glucose, lipids, and other metabolic signals (Yu et al., 2012; Enzo et al., 2015; Noto et al., 2017; Zhang et al., 2017). More interestingly, YAP regulates the activity of key enzymes involved in the serine pathway in breast cancer (Wu et al., 2017), and YAP-induced serine/glycine metabolism was reported to be regulated by phospholipase C epsilon (PLCε) in prostate cancer (PCa) (Duan et al., 2020). However, the relationship between YAP and serine metabolism in colon cancer remains unclear.

In this study, we constructed colon cancer cell models with serine deprivation resistance and found that YAP expression was increased in these models. An increase in serine concentration can also accelerate YAP expression, which, in turn, activates downstream signaling pathways and promotes cell proliferation. The molecular mechanism is as follows: a high serine concentration promotes the binding of YAP to the deubiquitinating enzyme USP7, thereby preventing the ubiquitin-mediated degradation of YAP. Therefore, inhibition of YAP activity can inhibit the proliferation of colon cancer cells and induce apoptosis. YAP inhibitors were also used to treat colon cancer organoids. A positive correlation between the expression of the deubiquitinating enzyme USP7, the YAP target gene CTGF, and the serine metabolic enzyme PHGDH was detected in colon cancer tissues. Our findings provide evidence for further understanding of the role of YAP in the serine metabolism of colon cancer and targeted treatment strategies.

## Materials and Methods

### Cell Culture

Human colon adenocarcinoma cells (SW620, SW480, HT29, HCT116, and LOVO) were purchased from the Shanghai Institute of Cell Research, Chinese Academy of Sciences (Shanghai, China). All cell lines were cultured in an RPMI-1640 medium (Thermo Fisher Scientific, Salem, MA, United States) supplemented with 10% (v/v) fetal bovine serum (Every Green, Hangzhou, China), 100 U/mL penicillin, and 100 μg/mL streptomycin (Thermo Fisher Scientific, Salem, MA, United States), and cultured in a humidified 5% CO_2_ incubator at 37°C. Serine-deficient tolerant cells, SW620-S and LOVO-S, were generated from the parental cell lines SW620 and LOVO, respectively, following persistent exposure to a serine-free medium (Zhao et al., 2020b) for approximately 1 month.

### Cell Proliferation Assay

#### CCK-8 Assay

Human colon adenocarcinoma cells (1 × 10^4^ cells/well) were seeded in 96−well plates with a 100 μL RPMI-1640 complete medium and incubated for 24 h at 37°C in an atmosphere containing 5% CO_2_. Subsequently, a complete medium was replaced with a medium containing different concentrations of serine for 24 h. Then, 10 μL of the CCK-8 reagent (Beyotime, Shanghai, China) was added to each well, and the plates were cultured at 37°C for 1 h continuously. The optical density (OD) value at 450 nm was measured by a microplate reader (Synergy HT ZX-22; Bio-Tek Instruments, VT, United States).

#### Colony Formation Assay

The cells were seeded in a 6-well plate (300 cells/well), cultured with YAP inhibitors in an incubator for 2 weeks at 37°C in an atmosphere containing 5% CO_2_. Cultivation was terminated when a colony was visible, and the colonies were washed three times with PBS. The washed colonies were stained with 4% paraformaldehyde for 10 min and subsequently with 0.1% crystal violet solution for 30 min. The number of colonies was counted using Image J, version 1.52 (NIH Image, Bethesda, MD).

### RNA-Sequencing Analysis

A TRIzol reagent (Carlsbad, CA, United States) was used to extract total RNA from SW620 cells and SW620-S cells treated with CBR5884 (MCE, Shanghai, China) or VP (MCE, Shanghai, China). Three independent replicates were analyzed. RNA-sequencing (RNA-seq) was performed by KAITAI-BIO (Hangzhou, China). RNA-seq libraries were constructed using the Illumina TruSeq RNA sample preparation kit (RS-122-2001) and sequenced using an Illumina high-seq 2000 with a read length of 50 bp with pair ends. RNA-seq reads were mapped to the human genome (hg19) using TopHat (Trapnell et al., 2009). Only those reads mapped to unique genomic locations and with < 5% mismatches were analyzed further. We used FPKM (Trapnell et al., 2010) to measure gene transcript express and DEGSeq (Wang et al., 2010) to identify differentially expressed genes. The differentially expressed genes were counted and annotated using the NCBI, UniProt, GO, KEGG, and GSEA databases to obtain detailed description information. These raw data were submitted to https://www.ncbi.nlm.nih.gov/sra/PRJNA682868 for analysis.

### Flow Cytometry Assay

SW620/SW620-S, SW480/SW480-S, LOVO/LOVO-S, and HT116/HT116-S cells cultured to the logarithmic growth phase were digested with EDTA-free trypsin (Gibco BRL, Grand Island, NY, United States), washed three times with PBS, and resuspended in a 195 μL FITC-conjugated Annexin V binding buffer (Beyotime, Shanghai, China). Subsequently, 2 μL of Annexin-V-FITC and 1 μL of propidium iodide were added to the mixture, which was then incubated for 15 min at room temperature (RT) in the dark. After gentle vortexing, the sample was analyzed by flow cytometry (Beckman Coulter, Fullerton, CA, United States).

### Quantitative Real-Time PCR (qRT-PCR)

Total RNA was extracted from cells by using a TRIzol Reagent (Carlsbad, CA, United States) according to the manufacturer’s instructions. Then, the reverse transcription reaction was performed with 1 μg of total RNA and a Quantscript RT kit (Takara, Osaka, Japan). mRNA expression was determined by quantitative real-time PCR using a Roche LightCycler^®^ 480II qPCR system (Roche, Basel, Switzerland). Gene expression was normalized to that of β-actin, and the relative quantification was calculated by the 2^−ΔΔCt^ method. The primers used are presented in Supplementary Table 1.

### Preparation of Cytoplasmic and Nuclear Extracts

The cells were harvested, washed once with PBS, resuspended in 200 μL of lysis buffer A (0.1% NP40, 1 × Roche Protease Inhibitors, 10 mM KCl, 1.5 mM KH_2_PO_4_, 0.14 M NaCl, 8 mM Na_2_HPO_4_), and left for 1 min on ice. Then, one-fifth of it was reserved as the total cellular protein. The remainder was centrifuged at 10,000 rpm for 30 s. The supernatant (representing the cytoplasmic extract) was collected, and the pellet (corresponding to the crude nuclei) was washed four times with 200 μL of lysis buffer A. A RIPA lysis buffer was added for immunoblotting analysis.

### Immunoblotting Analysis

The cell or tissue samples were lysed by a RIPA lysate buffer (Beyotime, Shanghai, China) containing 1 × protease inhibitor cocktail (Beyotime, Shanghai, China). Protein concentration was quantified using the BCA Protein Assay Kit (Beyotime, Shanghai, China). The samples (20 μg/lane) were separated by sodium dodecyl sulfate-polyacrylamide gel electrophoresis (SDS-PAGE), and the separated proteins were transferred to a PVDF membrane (BioRad, Hercules, CA, United States). Non-specific binding was blocked by incubation of the membranes in 5% defatted milk for 1 h at RT. The membranes were incubated overnight with primary antibodies at 4°C and with secondary antibody for 2 h at RT. After incubation, the membranes were washed three times with TBST. The ECL Kit (Fdbio, Zhejiang, China) was applied to the membrane, and the membrane was imaged using a ChemiDoc XRS + (BioRad, Hercules, California, United States). The gray intensity of the target band was analyzed by Image Lab software. The antibodies used are presented in Supplementary Table 2.

### Metabolomic Analyses

#### Sample Preparation of Cell Lysate for Metabolomic Analyses

For adherent cell culture, the medium was gently aspirated, and cells on the surface of the well plates were gently washed three times with ice-cold PBS. The bottom (outer wall) of the culture dish was contacted with liquid nitrogen to quench the cells. Then, 500 μL of methanol–water (4:1, v/v) was added, and adherent cells were scraped from the plate surface. Next, the cells were transferred to a 1.5 mL centrifuge tube, and 500 μL of methanol–water (4:1, v/v) was added to the Petri dish to transfer as many of the remaining cells to the tube as possible. Finally, the samples were sent to Dian Diagnostics for analysis.

#### UPLC-MS Analysis

Untargeted LC-MS analysis of metabolites was performed by using an HPLC system (HPLC-1220 Infinity II, Agilent) interfaced with a mass spectrometer (6545 Q-TOF, Agilent). Electrospray ionization (ESI) in the mass spectrometer was conducted in positive mode in full scan with a mass range of 100–1,700 m/z. Sheath and auxiliary gas flow were set at 11 L/min, with a capillary temperature of 370°C. The ESI source voltage and capillary voltage were 3.5 kV and 40 V, respectively. A pooled quality-control mixture comprising equal aliquots of all samples was run at regular intervals throughout each analytical batch. Samples were randomized for each analytical batch. Chromatographic separation was performed on an Agilent Eclipse Plus C18 (2.1 × 100 mm, 1.8 μm) column. The column and autosampler temperatures were maintained at 30 and 4°C, respectively. The mobile phase was composed of 0.1% formic acid water (A) and acetonitrile (B) at a flow rate of 0.3 mL/min. The gradient conditions were as follows: 0–2 min, 5% B; 2–20 min, 5–100% B; 20–25 min, 100% B; re-equilibration: 5 min. The injection volume was 2 μL.

#### Data Preprocessing and Metabolite Identification

Raw UPLC–MS data were preprocessed and analyzed with Agilent Mass Hunter Qualitative Navigator B.08.00 and Profinder 10.0. The pooled quality control mixture was used for signal correction in the between-batch and within-batch analyses. The samples were normalized based on their cell count. The resultant data matrices were imported into the SIMCA14.1 (Umetrics, Umeå, Sweden) software for multivariate statistical analysis. Orthogonal partial least-squares discriminant analysis (OPLS-DA) and partial least-squares discriminant analysis (PLS-DA) were used for metabolite profile analysis. The identities of marker metabolites were verified by comparison of their retention time and mass spectra with those of commercially available standards. Pathway analysis was performed using MetaboAnalyst 3.0^1^.

### Gene Transfection and RNA Interference

The siRNA primers were obtained from RIBOBIO; the following sequences were used: siRNA-USP7 #1, GAATGACA TGTACGATGAA; siRNA-USP7 #2, GAGCGACCTTACCC AAGTT; and siRNA-USP7 #3, TAAGGACCCTGCAAATTAT.

To silence USP7 in colon cancer cells, siRNAs were transfected at a final concentration of 50 nM using Lipofectamine^®^ RNAiMAX (Invitrogen, CA, United States) in accordance with the manufacturer’s recommendations.

The Flag-YAP and HA-USP7 plasmids were obtained from Sino Biological (Beijing, China). All plasmids were transfected into 293T cells using Lipofectamine 2000 for experiments.

### Co-IP Assay

Briefly, cells were lysed with an IP lysis buffer (Beyotime, Shanghai, China), and the protein concentration was quantified by a BCA Protein Assay Kit (Beyotime, Shanghai, China). Co-IP assays were performed using a Pierce Direct Magnetic IP/Co-IP kit (Thermo Fisher Scientific, Rockford, United States) in accordance with the manual. The proteins were eluted in a 2 × loading sample buffer and then boiled for 10 min at 100°C. The subsequent detection steps were as described for immunoblotting analysis. The antibodies used are presented in Supplementary Table 2.

### Human Colon Tumor Organoid Culture

Tumor tissue was minced and digested with collagenase I (1 mg/mL, Sigma-Aldrich, Shanghai, China) and dispase (0.6 mg/mL, Sigma-Aldrich, Shanghai, China) for 15 min at 37°C, embedded in RGM BME type 2 Matrigel, and cultured in a human complete medium [AdDMEM/F12 medium supplemented with HEPES (10 mM), GlutaMAX (2 mM), epidermal growth factor (EGF, 50 ng/mL), fibroblast growth factor 10 (FGF10, 10 ng/mL), noggin (100 ng/mL), Y-27632 (10 μM), A8301 (0.5 μM), all from Sigma-Aldrich, and penicillin/streptomycin (1 ×, Thermo Fisher Scientific, MA, United States)].

### Hematoxylin-Eosin (H&E) Staining

To process the organoids, the matrix gel was digested with 1 mg/mL dispase and washed three times with PBS. The organoids were then fixed with 10% formalin. The procedure, including H&E staining, was performed by our authors in the Department of Pathology, Affiliated Jinhua Hospital, Zhejiang University School of Medicine.

### Immunohistochemistry

Anti-CDX2 and anti-SATB2 antibodies from Abcam were used for immunohistochemical analyses. Immunohistochemistry was performed by our authors in the Department of Pathology, Affiliated Jinhua Hospital, Zhejiang University School of Medicine.

### Statistical Analysis

The GraphPad Prism 6.0 software (GraphPad, San Diego, CA, United States) was used for data analysis. The values of IC_50_ and HillSlope were analyzed by applying the equation log(inhibitor) vs. response—Variable slope. All data were recorded as the mean ± SD. Statistical significance of results was determined from the Student’s *t*-test, with *p*-values of < 0.05 regarded as statistically significant.

## Results

### Serine Starvation–Resistant Colon Cancer Cell Models

**FIGURE 1:**
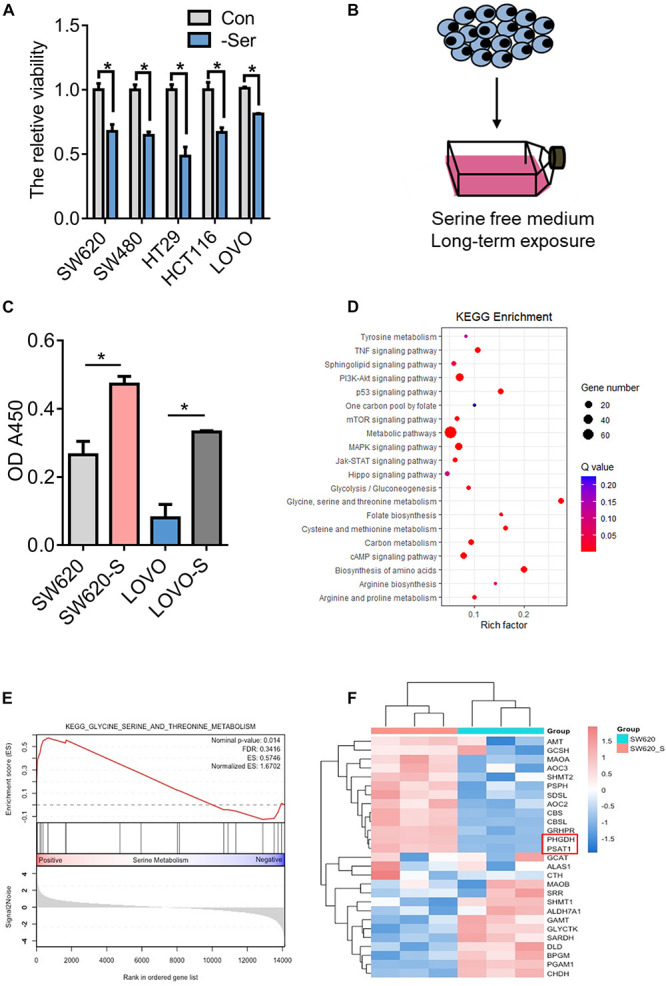
Generation and characterization of serine-starvation-resistant cells. **(A)** CCK-8 analysis of cell viability in colon cancer cells (SW620, SW480, HT29, HCT116, and LOVO) cultured in basic and serine-deprived medium. Error bars represent mean ± SD (*n* = 3). *p < 0.05. **(B)** Schematic of the generation of serine-starvation-resistant cells. **(C)** The proliferation ability of the SW620, SW620-S, LOVO, and LOVO-S cells under the condition with serine or without serine measured by CCK-8. Error bars represent mean ± SD (*n* = 3). **(D)** KEGG annotations and enrichment of differentially expressed genes (*Q*-value) in SW620-S cells compared to SW620. Nodes in red indicate significance (*Q* < 0.05), and the size of the nodes indicates gene number. **(E)** Glycine, serine, and threonine metabolism gene set enrichment analysis (GSEA) of RNA-seq data in SW620-S relative to SW620 cells. **(F)** Heat map of significantly changed genes involved in glycine, serine, and threonine metabolism pathway.

Serine is essential for cell proliferation. It is known that serine deprivation can significantly inhibit the proliferation of HCT116 colon cancer cells (Maddocks et al., 2013, 2016, 2017; Ducker and Rabinowitz, 2017). In this study, we used a serine-free culture medium and found that the proliferation of many colon cancer cells was inhibited (Figure 1A). However, serine deprivation did not cause a significant increase in cell apoptosis (Supplementary Figure 1A). Serine is a non-essential amino acid, which can be obtained through exogenous uptake and endogenous synthesis (Supplementary Figure 2A). We cultured two types of colon cancer cells, SW620 and LOVO, in a medium deprived of serine for about 2 weeks; these are referred to as SW620-S and LOVO-S, respectively (Figure 1B). We found that the proliferation ability of cells obtained from long-term exogenous serine deprivation was stronger than that of parental cells (Figure 1C), suggesting that these cells were resistant to serine deprivation.

We then performed transcriptome profiling by RNA-Seq in SW620-S cells to gain insight into the molecular basis of serine deprivation resistance. In total, 1116 differential genes were identified: 429 genes were upregulated and 687 were downregulated [fold change (FC) > 2, FDR < 0.05, Student’s *t*-test] (Supplementary Table 3). KEGG and GSEA analysis revealed that the glycine, serine, and threonine metabolism pathway was upregulated (Figures 1D,E). SSP is catalyzed by PHGDH, PSAT1, and PSPH (Supplementary Figure 2A). Here, PHGDH and PSAT1 were increased in SW620-S cells compared with SW620 (Figure 1F), and we confirmed the mRNA and protein expression of these genes in SW620/SW620-S and LOVO/LOVO-S (Supplementary Figures 2B,C). These studies revealed that the enzymes involved in the *de novo* serine synthesis pathway were upregulated dramatically.

We further analyzed the metabolomics of resistant cells. We employed a comparative metabolomics approach to yield profiles for SW620-S and SW620 cells that were segregated independently by OPLS-DA analysis, illustrating that each population is metabolically obvious. Approximately 132 distinct metabolites from diverse chemical classes were detected (Supplementary Figures 2D,E). KEGG analysis of the significantly altered metabolites enriched glycerophospholipid metabolism, glycine, serine, and threonine metabolism, purine metabolism, and sphingolipid metabolism pathways (Supplementary Figure 2F).

To examine the hypothesis that increased PHGDH meets the requirement of cells cultured in serine-depleted media for proliferation, we treated cells with CBR5884, an inhibitor of PHGDH. Compared with SW620-S cells, the proliferation of SW620-S cells treated with CBR5884 was impeded significantly (Supplementary Figure 3A), and a distinctly different metabolite profile was revealed, which included 70 metabolites (Supplementary Figures 3B–D). KEGG analysis of the significantly altered metabolites enriched glycerophospholipid metabolism, purine metabolism, and sphingolipid metabolism pathways, which have been changed (Supplementary Figure 3E). Collectively, the results of the transcriptomics and metabolomics studies showed that *de novo* serine synthesis was essential in serine deprivation-resistant cells.

### Short-Term and Long-Term Deprivations of Serine Have Opposite Effects on the Regulation of YAP Expression

We studied the signaling pathways involved in the resistance process. As shown in Figure 1D, there are many signaling pathways enriched, including the TNF signaling pathway, the PI3K-Akt signaling pathway, the p53 signaling pathway, the mTOR signaling pathway, the MAPK signaling pathway, the Jak-STAT signaling pathway, and the Hippo signaling pathway. Many studies have demonstrated the regulation between serine and those signaling pathways (Maddocks et al., 2013; Zhai et al., 2015; Pathria et al., 2018; Kim et al., 2019; Duan et al., 2020). YAP is the key molecule in the Hippo signaling pathway, and the overexpression of YAP is closely linked to high cell proliferation, metastasis, and poor survival of patients with colon cancer (Wang et al., 2013; Song et al., 2018). Therefore, we focused on the Hippo signaling pathway for further study.

**FIGURE 2:**
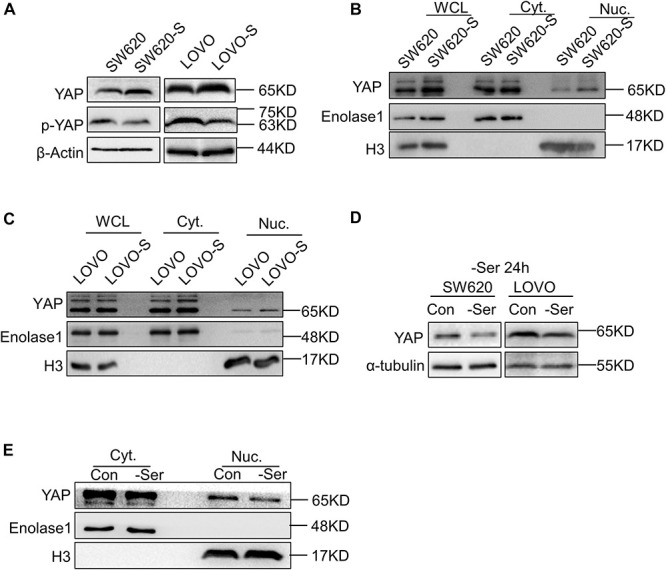
Short-term and long-term deprivations of serine have opposite effects on the regulation of YAP expression. **(A)** Immunoblotting analysis of YAP and p-YAP protein expression in SW620 and SW620-S, LOVO, and LOVO-S cells. **(B,C)** Analysis of cellular localization of YAP in SW620, SW620-S, LOVO, and LOVO-S cells. Immunoblotting analysis of YAP in Nuc. and Cyt. separation. Enolase 1 and H3 are markers for cytoplasm and nuclear, respectively. Cyt., Cytoplasm; Nuc., nucleus. **(D)** Immunoblotting analysis of YAP protein expression in SW620 and LOVO cultured under a medium with or without serine. **(E)** Nucleocytoplasmic separation analysis of YAP cellular localization change in SW620 cells cultured with or with serine contained medium for 24 h. Immunoblotting analysis of Nuc. and Cyt. separation with the indicated antibodies.

To explore the role of YAP in serine metabolism, we examined the expression of YAP and p-YAP in SW620-S and LOVO-S cells. The upregulation of YAP and the downregulation of p-YAP were detected in serine starvation–resistant cells compared with parental cells (Figure 2A). YAP exerts transcriptional activation by entering the nucleus. Therefore, the expression of YAP in the nucleus represents the degree of activation of this signaling pathway (Meng et al., 2016). We used cytoplasmic nuclear separation technology to detect YAP expression in the nucleus. As tubulin protein expression has been reported in the nucleus (Schwarzerová et al., 2019), we switched to a glycolysis-related enzyme, enolase 1, as a cytoplasmic protein marker. The expression of enolase 1 is not affected by serine deprivation (Supplementary Figure 4A). The expression of YAP in the nucleus of resistant cells was significantly increased (Figures 2B,C). Consistently, the mRNA expression of the YAP target genes CTGF, CDX2, and CYR61 was elevated in SW620-S cells (Supplementary Figure 4B). These results suggested that YAP expression was increased in serine starvation–resistant cells, which activated the downstream signaling pathway.

Interestingly, when the parental cells SW620 and LOVO were cultured transiently (e.g., for 24 h) in serine-free conditions, the overall expression and nuclear YAP expressions were both decreased (Figures 2D,E).

Collectively, these results indicated that short-term and long-term deprivation of serine have opposite effects on the regulation of YAP expression.

### Serine Deubiquitinates YAP by Promoting the Interaction Between USP7 and YAP

**FIGURE 3:**
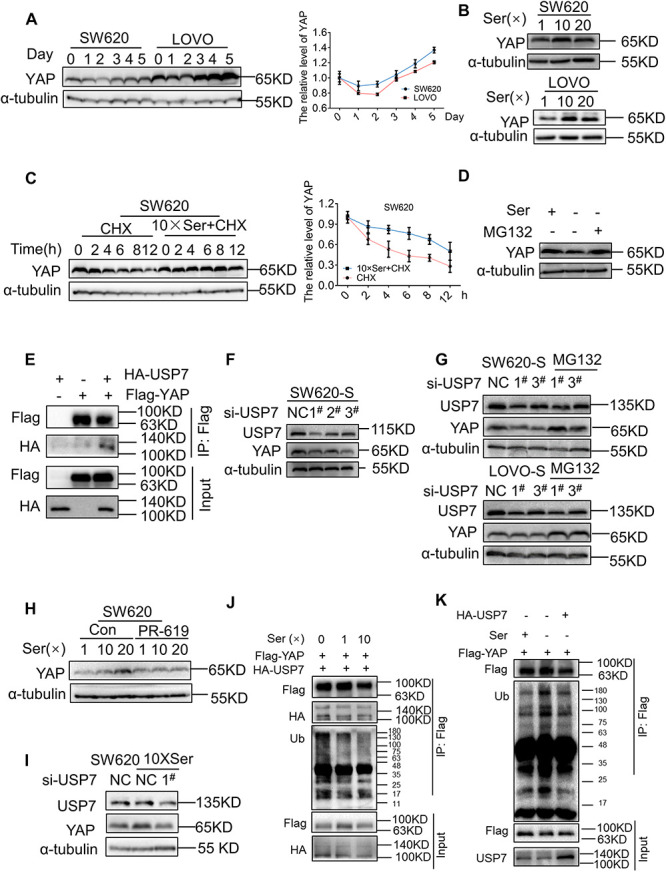
Serine deubiquitinates YAP by promoting the interaction between USP7 and YAP. **(A)** SW620 and LOVO were cultured with a medium without serine and harvested at the indicated days; protein levels of YAP were analyzed by immunoblotting (left). Quantification of YAP protein levels for each point was determined by densitometry (right). **(B)** SW620 and LOVO cells were treated with high concentration of serine. Immunoblotting analysis was performed with the indicated antibodies. The concentrations of 1, 10, and 20 × Ser are 30, 300, and 600 μg/mL, respectively. **(C)** SW620 cells were treated with cycloheximide (40 μg/mL) under a basis culture medium and a medium containing 10 × Ser, and harvested at the indicated times; protein levels of YAP were analyzed by immunoblotting (left). Quantification of YAP protein levels for each point was determined by densitometry (right). **(D)** SW620 cells cultured with a medium without serine were left untreated or treated with MG132 (10 μM) for 6 h, followed by cell lysates being immunoblotted as indicated. **(E)** Interaction between exogenous USP7 and YAP. 293T cells were co-transfected with indicated constructs. Cellular extracts were immunoprecipitated with magnetic beads and FLAG antibody. Immunoprecipitations were performed with antibodies against the indicated proteins. **(F)** Immunoblotting analysis of YAP protein expression in SW620-S treated with si-USP7 (1^#^, 2^#^, 3^#^) **(G)** SW620-S and LOVO-S transfected with indicated siRNA were treated with or without MG132 (10 μM) for 6 h, followed by immunoblotting analysis being performed with the indicated antibodies. **(H)** YAP protein expression in SW620 treated with 10 μM PR-619 under different concentrations of serine was detected by immunoblotting. **(I)** SW620 cells cultured with 10 × Ser (600 μg/mL) were treated with si-USP7 1^#^. Immunoblotting analysis was performed with the indicated antibodies. **(J)** 293T cells co-transfected with Flag-YAP and HA-USP7 were cultured with different concentrations of serine all the time and treated with MG132 (10 μM) for 6 h before harvest. Cell lysates were immunoprecipitated with magnetic beads and FLAG antibody to detect the ubiquitin chains on YAP. **(K)** 293T cells transfected with Flag-YAP or co-transfected with Flag-YAP and HA-USP7 were cultured with or without serine all the time and treated with MG132 (10 μM) for 6 h before harvest. Cell lysates were immunoprecipitated with magnetic beads and FLAG antibody to detect the ubiquitin chains on YAP.

We studied the mechanism for the regulation of YAP expression following different periods of serine deprivation. YAP underwent a significant decrease in the first 2 days of serine withdrawal; this was followed by a clear increase in protein expression (Figure 3A). CTGF expression followed the changes in YAP (Supplementary Figure 5A). After deprivation of exogenous serine, the cell activates the endogenous synthesis pathway as a means of compensation. PHGDH in parental cells was gradually upregulated over time after serine deprivation, suggesting an increase in endogenous serine synthesis (Supplementary Figure 5B). Therefore, we proposed that the cause of the different effects on YAP expression following short-term and long-term deprivation of serine may be the fluctuation in serine concentrations.

To test this, the parent cells were subjected to short-term exposure in a medium with increased serine concentration. For both SW620 and LOVO cells, the expression of YAP and target genes was improved significantly following incubation in a medium with high serine content. However, no further increase in YAP expression was seen following incubation with 20 × Ser compared with 10 × Ser (Figure 3B and Supplementary Figures 5C,D). These results indicated that serine promoted YAP expression. Short-term deprivation of serine leads to a reduction in the serine content and YAP expression. Therefore, long-term deprivation leads to an increase in endogenous serine synthesis, thereby increasing the expression of YAP. Next, we studied the mechanism by which serine regulates YAP. Changes in serine concentration did not affect the mRNA expression of YAP (Supplementary Figure 5E), suggesting that it was not regulated at the transcriptional level but may occur post-transcriptionally. Cells cultured in a medium with normal or high serine content were treated with cycloheximide (CHX), a general inhibitor of protein synthesis. The subsequent half-life analyses showed that the YAP protein was much more stable in cells cultivated with a high concentration of serine (Figure 3C). As YAP was shown to experience proteasomal degradation activated by upstream kinases (Yan et al., 2020), cells were treated with the proteasome inhibitor MG132, and the reduction in YAP protein expression by serine deficiency was restored (Figure 3D and Supplementary Figure 5F). These results suggested that the regulation of YAP by serine involved in the regulation of protein ubiquitination.

Many studies have reported the protective effect of deubiquitinating enzymes on YAP protein (Kim and Sixma, 2017; Sun et al., 2019; Zhu et al., 2020). To test whether deubiquitinase regulated YAP stability in serine metabolism, we treated cells with PR619 (an inhibitor of USP4, USP8, USP7, USP2, and USP5) and degrasyn (an inhibitor of USP9x, USP5, USP14, and UCH37), two generalized DUB inhibitors. PR619, but not degrasyn, promoted the degradation of YAP (Supplementary Figure 5G). USP7 is a deubiquitinating enzyme of YAP, and PR619 can inhibit USP7. Therefore, we hypothesized that USP7 was involved in the regulation of YAP by serine content. Consistently, YAP indeed physically interacted with USP7, as shown by Co-immunoprecipitation (Co-IP) assay after co-transfection of exogenous YAP and USP7 into 293T cells (Figure 3E). We found that YAP, CTGF, and CDX2 were downregulated after treatment with siRNA to USP7 in SW620-S cells (Figure 3F and Supplementary 5H). Next, we treated USP7-silenced cells with MG132 and found that the protein expression of YAP was restored (Figure 3G). Furthermore, the inhibition of USP7 with PR619 or siRNA to USP7 blocked the high expression of YAP caused by serine (Figures 3H,I).

Moreover, the interaction between YAP and USP7 was enhanced by high serine (Figures 3J,K). These results indicated that serine promoted the expression of YAP by strengthening the interaction between USP7 and YAP.

### YAP Enhances the Proliferation of Serine Starvation–Resistant Colon Cancer Cells

**FIGURE 4:**
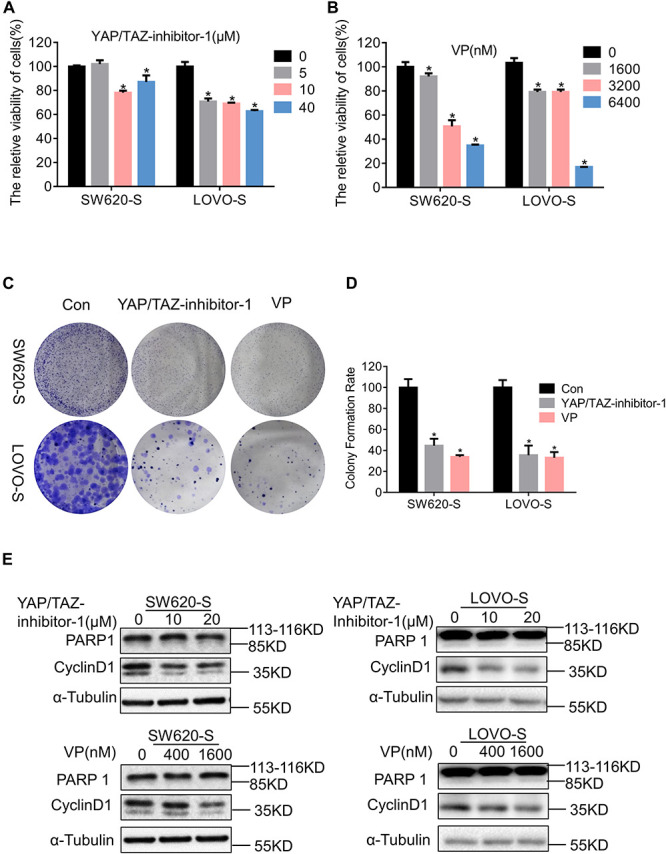
YAP enhances the proliferation of serine starvation–resistant colon cancer cells. **(A,B)** Cell viability of SW620-S and LOVO-S treated with different concentrations of YAP/TAZ-inhibitor-1 or Verteporfin (VP) were analyzed by CCK-8. Error bars represent mean ± SD (*n* = 3). *p < 0.05. **(C,D)** The cell proliferations of SW620-S and LOVO-S treated with YAP/TAZ-inhibitor-1(20 μM) or Verteporfin (VP) (500 nM) were measured by colony formation assay. Error bars represent mean ± SD (*n* = 3). *p < 0.05. **(E)** SW620-S and LOVO-S cells were treated with different concentrations of YAP/TAZ-inhibitor-1 or Verteporfin (VP). Immunoblotting analysis was performed with the indicated antibodies. 85 KD was cleaved from PARP.

We used two inhibitors of YAP, YAP/TAZ-inhibitor-1 and VP, to study the function of YAP in serine starvation–resistant cells. Both short-term cell viability testing and long-term clone formation experiments showed that YAP inhibition significantly inhibited the proliferation ability of serine starvation–resistant cells (Figures 4A–D). At the molecular level, we discovered that CyclinD1 was significantly decreased by the YAP inhibitor (Figure 4E). However, PARP1 did not undergo significant cleavage (Figure 4E), suggesting that YAP generally maintained the proliferation of serine starvation–resistant cells rather than resisting cell apoptosis. We further analyzed the transcriptome and metabolome of serine starvation–tolerant cells after YAP inhibition. Transcriptome sequencing found 1711 upregulated genes and 2607 downregulated genes (Supplementary Figures 6A,B). KEGG analysis found that the proteasome signal pathway was enriched (Supplementary Figure 6C). Interestingly, the expression of many USP genes, including USP7, was downregulated after YAP inhibition (Supplementary Figure 6D), suggesting that there may be a positive feedback loop between YAP and USP7.

Relative changes in 147 metabolites were detected in SW620-S cells treated with VP; notably, metabolites in the sphingolipid metabolism, glycine, serine and threonine metabolism, and glycerophospholipid metabolism pathways were enriched in the KEGG analysis (Supplementary Figures 6E–H). The metabolomics results suggested that YAP may regulate serine metabolism. Therefore, there may be a bidirectional regulation between serine metabolism and YAP expression.

### YAP Inhibitor Modulates Cell Phenotype and Proliferation in Colon Cancer Organoids

To determine the clinical relevance of our experimental findings, we analyzed TCGA RNA-Seq data. YAP was overexpressed in 41 colon cancer tissues compared with paired normal tissues (Supplementary Figure 7A), and the expression of YAP was negatively correlated with overall survival (OS) in patients with colon cancer (Supplementary Figure 7B). However, there was no correlation between the expression of YAP and the stage of colon cancer (Supplementary Figure 7C). Next, we verified the expression of YAP in cancer tissue and paired para-carcinoma tissue from 10 patients with colorectal cancer. In line with the above findings, we discovered six tumor tissues with higher YAP expression relative to paired para-carcinoma tissues, one tumor tissue with lower YAP expression, and three tumor tissues with no significant change (Supplementary Figures 7D,E).

**FIGURE 5:**
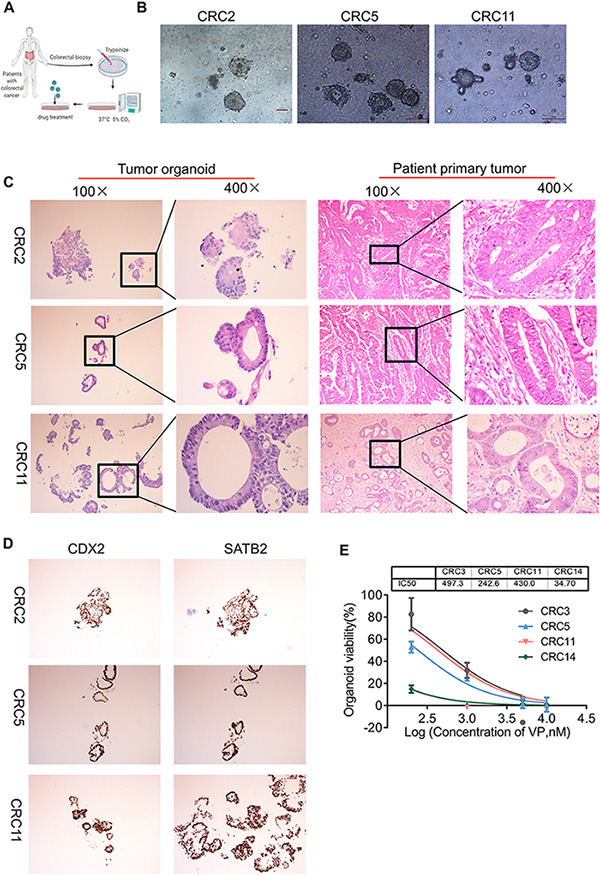
YAP inhibitor modulates cell phenotype and proliferation in colon cancer organoids. **(A)** Key procedures for generating colon cancer organoid cultures. **(B)** Morphological observation of organoids derived from three different patients with colon cancer. Scale bars, 100 μm. **(C)** Hematoxylin and eosin (H&E) images showing the tumor organoid phenotype (left two columns: 100 and 400×) and patient primary tumor architecture (right two columns: 100 and 400×). **(D)** Immunohistochemistry (IHC) analysis of colon cancer marker gene CDX2 and SATB2 in colon tumor organoids (100×). **(E)** The viability of organoids treated with Verteporfin (VP) was determined by CCK-8. The IC_50_ of VP in organoids derived from different patients was indicated above. Error bars represent mean ± SD (*n* = 3).

Organoid is a cogent vehicle for precision medicine and drug screening (Aboulkheyr Es et al., 2018; Jin et al., 2018). To investigate the effect of VP on human colon cancers, we successfully modeled organoids from patients with colon cancer in a medium supplemented with the growth factors noggin, epidermal growth factor (EGF), human fibroblast growth factor 10 (FGF-10), HEPES, GlutaMAX, Y-27632, and A83-01 (Figure 5A). Colon organoids in the culture conditions presented as irregular compact structures (Figure 5B) and resembled the primary tumor morphology (Figure 5C). Notably, CDX2, one of the YAP target genes that is an intestinal-specific transcription factor that regulates the regeneration and differentiation of intestinal epithelial cells, as well as SATB2, a highly sensitive and specific marker for highly differentiated neuroendocrine tumors at the rectal/straight B junction, were expressed in tumor organoids as revealed by the immunohistochemistry analysis (Figure 5D).

We then explored whether the antiproliferative effect of VP was also observed in colon organoids. The four organoids, CRC3, CRC5, CRC11, and CRC14, were derived from patients with poorly differentiated colonic adenocarcinoma, moderately differentiated adenocarcinoma of the rectum, moderately differentiated colonic adenocarcinoma, and hepatic metastasis of colonic carcinoma, respectively. VP treatment suppressed the viability and impaired the formation of organoids, from dense to loose (Figure 5E and Supplementary Figure 8A). The IC_50_ of VP in CRC3, CRC5, CRC11, and CRC14 was 497.3, 242.6, 430.0, and 34.70 nM, respectively. YAP was more highly expressed in colon carcinomas with metastases compared with colon carcinomas without metastases (Supplementary Figure 8B). Consistent with this, the organoids derived from colon carcinomas with metastases were sensitive to YAP inhibitors. Together, these experimental results provide evidence that colon organoids depending on YAP were sensitive to VP, which may indicate a potential drug target.

### Positive Correlation Between PHGDH, USP7, and CTGF mRNA in Patients With Colon Cancer

**FIGURE 6:**
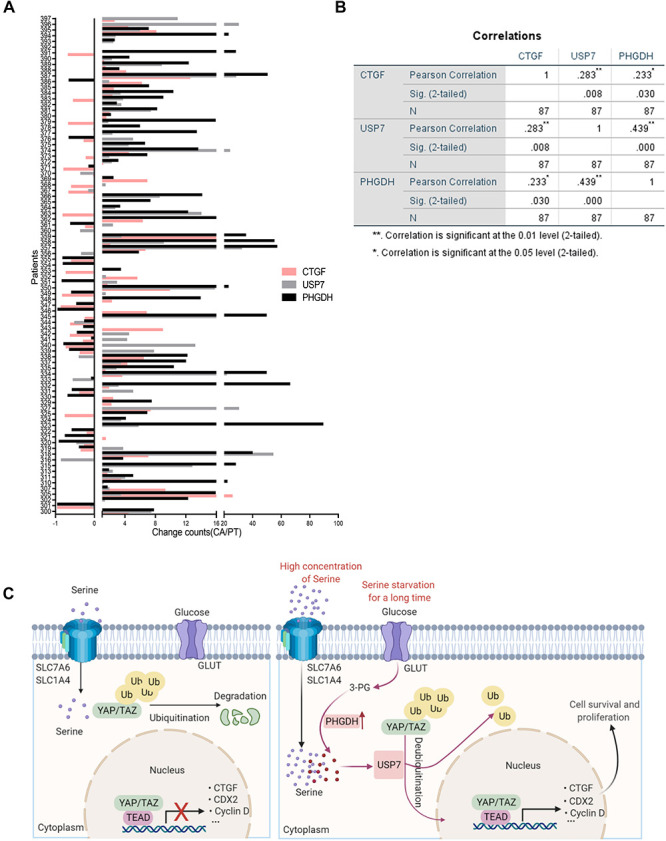
The positive correlation between PHGDH, USP7, and CTGF mRNA in colon cancer patients. **(A)** Samples were obtained from 87 patients with colon cancer. qRT-PCR analysis of paired samples of para-carcinoma region (N) and tumor region (T) from the same patient. **(B)** Correlations of expression between USP7 and CTGF, PHGDH and USP7 in the 87 colon cancer patients were performed (*n* = 87). *p < 0.05; **p < 0.01. **(C)** Scheme for the regulatory mechanism of YAP under different conditions.

To further explore the relationship between PHGDH, USP7, and CTGF, we evaluated the mRNA expression of PHGDH, USP7, and CTGF in tissues from 87 patients with colon cancer via qRT-PCR. Most tumor specimens showed higher expression of PHGDH, USP7, and CTGF compared with the matched para-carcinoma tissues (Figure 6A). Furthermore, statistically significant correlations were found between PHGDH and USP7 or CTGF, USP7, and CTGF expression (Figure 6B, PHGDH and USP7, *r* = 0.439, *p* < 0.001; PHGDH and CTGF, *r* = 0.233, *p* < 0.05; USP7 and CTGF, *r* = 0.283, *p* < 0.01; *n* = 89). Collectively, these results validated our hypothesis that YAP expression can be enhanced by USP7 when the *de novo* serine synthesis pathway is activated.

## Discussion

Dysregulation of serine metabolism is closely correlated with the occurrence and development of tumors (Mattaini et al., 2016; Maddocks et al., 2017). In many cases, extracellular serine alone can supply cancer cells with sufficient energy for proliferation; however, in some cancer cells, increased glucose induces serine synthesis after cellular transformation to a malignant state or after the expression of specific oncogenes, or in response to alterations in nutrient availability and tissue environment (Cairns et al., 2011; Davidson et al., 2016; Hensley et al., 2016; Mullarky et al., 2016). Our results provide the first indication that the activity of SSP and the Hippo signaling pathway were increased in cells in response to long-term serine deficiency.

YAP and TAZ are two homologous transcriptional coactivators that promote cell proliferation, stem cell maintenance, and tissue homeostasis (Moon et al., 2018). Previously, cell density (Ou et al., 2020), cell polarity (Matakatsu and Blair, 2012), mechanical cues (Chen et al., 2015), cell–cell contact (Zhao et al., 2007), a large number of G-protein-coupled receptor (GPCR) ligands (Zhou et al., 2015), endoplasmic reticulum (ER) stress (Hong et al., 2017), osmotic stress (Lin et al., 2017), growth factors (Yu et al., 2015), and cellular energy status (Mo et al., 2015) have been shown to control YAP and TAZ. Under advantageous conditions, YAP and TAZ contribute to cell growth through a transcriptional program mediated by the TEAD family of transcription factors. As cellular energy and metabolites are necessary for survival and growth, YAP and TAZ are suppressed when the energy level of cells is low. Indeed, glucose, fatty acids, hormones, and other metabolic factors have been recently revealed to regulate YAP and TAZ (Cosset et al., 2017; Zhang et al., 2017; Koo and Guan, 2018). In contrast, some types of metabolic regulation, like glycolysis, lipogenesis, and glutaminolysis, are promoted by YAP and TAZ (Du et al., 2018; Koo and Guan, 2018; Zhang et al., 2018). Recently, Duan and his colleagues verified that YAP activity was also regulated by serine/glycine metabolism in a bidirectional manner. High YAP expression promotes serine/glycine production, and increased serine/glycine metabolism prevented YAP degradation by dephosphorylation (Duan et al., 2020). We found that short-term serine deficiency inhibits YAP activity, whereas, over longer periods, the adaptive response induces nuclear accumulation and activation of YAP, similar to the effects of ER stress (Wu et al., 2015) and osmotic stress (Hong et al., 2017; Lin et al., 2017). Our results also revealed the dynamic changes in YAP expression and activity that accompanied high PHGDH expression. These observations support the fact that cell growth and proliferation are dependent on the cellular energy level. Therefore, we hypothesized that the activation of YAP may be related to serine availability, whether from extracellular or intracellular synthesis. In accordance with the response to prolonged deprivation, YAP was also activated by the addition of extra serine, which supported our hypothesis.

Ubiquitination, a dynamic protein modification, can be inversely regulated by DUBs to reproduce free ubiquitin and alter the abundance and transcriptional activities of its substrate (Lei et al., 2017). Interestingly, various DUB inhibitors have been designed to target processes relevant to the maintenance of cancer and cancer stem cells (Kaushal et al., 2018). Ubiquitin-specific proteases (USPs) have been identified as one family of approximately 100 DUBs (Mevissen and Komander, 2017). USP9X was first found to regulate YAP via direct or indirect mechanisms as a DUB of AMOTL2, LATS, or YAP in different cancers (Kim et al., 2016; Li et al., 2018; Zhu et al., 2018). The abundance of YAP and TAZ was potently increased by USP10, which removed their poly-ubiquitin chains directly in HCC (Zhu et al., 2020). Evidence has accumulated to show that USP7 plays opposing roles in tumorigenesis because its function is dependent on context (Li et al., 2002; Zhan et al., 2017). For example, USP7 stabilizes the p53 protein to act as a tumor suppressor (Li et al., 2002) but promotes the proliferation of HCC cells via deubiquitination of YAP (Sun et al., 2019). In this study, we found that high concentrations of serine stabilized YAP; therefore, to gain insight into the regulatory mechanism, we focused on the relationship between USP7 and YAP in colon cancer models with abnormal serine metabolism. The Co-IP results obtained in this study showed that USP7 interacts with YAP, and that the high concentration of serine enhanced the deubiquitination function of USP7. The results showed that downregulation of USP7 by siRNA or an inhibitor could successfully suppress the expression and activation of YAP. Unexpectedly, the reduction in YAP protein induced by siRNA to USP7 was reversed by MG132. Collectively, our findings showed that abnormal serine metabolism stabilized YAP through USP7 to activate the Hippo pathway output, which stimulated tumor cell proliferation.

Activated YAP modulates various cellular functions, such as proliferation and apoptosis, which induces tissue expansion and tumorigenesis in many tissues through elevation of target gene expression (Schlegelmilch et al., 2011; Zhou et al., 2011; Hong and Guan, 2012). Hence, we treated serine-deficient colon cancer cells (SW620-S and LOVO-S) with VP, and experimental data showed that cell proliferation and CyclinD1 expression were dramatically reduced; however, there was no significant effect on apoptosis. The proteasome pathway and the glycine, serine, and threonine metabolism pathway were significantly enriched, as shown by RNA-Seq and metabolomics, respectively. Importantly, USP7 was downregulated by VP treatment. Moreover, in colon cancer tissues from patients, YAP was positively related by PHGDH and USP7. Therefore, serine metabolism and regulation of YAP expression may constitute a positive feedback loop.

As an organoid is derived from a specific organ, it can regenerate the physiology of its origin. Organoids are a powerful vehicle for diverse applications, including diagnosis and personalized medicine; here, organoids derived from patients with colon cancer have been used to study VP efficacy in human colon cancer cells. The results showed that VP inhibited the organoid activity, and the response of different patient-derived organoids to VP was interesting; in particular, colon carcinomas with metastases were more sensitive to YAP inhibition. However, owing to the lack of *in vivo* verification, this evidence is not suitable to shape clinical practice. Therefore, in the future, we aim to establish patient-derived xenograft models using colon cancer organoids for further study.

Ultimately, the results of our study showed that YAP promoted the growth and proliferation of colon cancer cells, and that its expression and activity were regulated by serine metabolism. In the future, we aim to explore the therapeutic effect of the targeted inhibition of YAP in combination with the regulation of serine metabolism on patients with YAP-dependent colon cancer. This will require an in-depth understanding of the overall metabolic state and cellular microenvironment of the tumor. Our current study did not examine the changes in nutrients in colon cancer tissues and para-cancerous tissues.

## Conclusion

YAP is degraded to maintain homeostasis under normal growth conditions. Our results demonstrated that an increase in serine concentration could cause USP7 to activate YAP by removing the poly-ubiquitin chain, therefore promoting cell survival and proliferation (Figure 6C). More specifically, treatment with VP could effectively suppress the expression of USP7 and tumor growth, which should provide clinical benefit for the treatment of colon cancer.

## Data Availability Statement

The datasets presented in this study can be found in online repositories. The names of the repository/repositories and accession number(s) can be found in the article/ Supplementary Material.

## Ethics Statement

Written informed consent was obtained from the individual(s) for the publication of any potentially identifiable images or data included in this article.

## Author Contributions

XZ and JFu performed the major experiments and analyzed most of the experimental data and wrote the manuscript. BH and JFa performed the organoid and histopathological experiments. LC, HL, and KP analyzed the metabolomic and transcriptome data. CG and JW performed colon cancer tissue collection. LF and LW performed qRT-PCR and immunoblotting experiments. XZ, JFu, BH, JFa, LC, HL, KP, CG, JW, LF, LW, JD, and WX provided assistance with all experiments and data interpretation. JD and WX supervised the entire project and designed all experiments. All authors reviewed the manuscript.

## Conflict of Interest

The authors declare that the research was conducted in the absence of any commercial or financial relationships that could be construed as a potential conflict of interest.

## Footnotes

1 http://www.MetaboAnalyst.ca/

## Abbreviations

YAP: Yes-associated protein
TAZ: transcriptional co-activator with PDZ-binding
VP: Verteporfin
TCA: tricarboxylic acid
NEAAs: non-essential amino acids
SSP: serine synthesis pathway
PHGDH: phosphoglycerate dehydrogenase
PSAT1: phosphoserine aminotransferase 1
PSPH: phosphoserine phosphatase
RT: room temperature
PLC ε: phospholipase C epsilon
PCa: prostate cancer
KEGG: Kyoto Encyclopedia of Genes and Genomes
EGF: epidermal growth factor
FGF-10: human fibroblast growth factor 10
HEPES: 2-[4-(2-hydroxyethyl)-1-piperazinyl] ethanesulfonic acid
TAZ: transcriptional coactivator with PDZ-binding
ER: endoplasmic reticulum
GPCR: G-protein -coupled receptor ligands
DUBs: deubiquitinating enzymes
USPs: ubiquitin-specific proteases.
