# Supplementary material for: Serine Metabolism Regulates YAP Activity Through USP7 in Colon Cancer
Source: Front Cell Dev Biol. 2021 May 12;9:639111. doi: 10.3389/fcell.2021.639111 (PMC8152669; doi:10.3389/fcell.2021.639111)
Supplement: Supplementary file 1 [file Table_1.DOC]

**Supplemental Table 1. Primers of qRT-PCR assay**

| Genes | Names | Primers (5’ to 3’) |
| --- | --- | --- |
| PHGDH | PHGDH-F | AACCGCAGCTTCTTGGCTTA |
|  | PHGDH-R | TAAGGCCTTCACAGTCCTGC |
| PSAT1 | PSAT1-F | ACTTCCTGTCCAAGCCAGTGGA |
|  | PSAT1-R | CTGCACCTTGTATTCCAGGACC |
| PSPH | PSPH -F | GACAGCACGGTCATCAGAGAAG |
|  | PSPH -R | CGCTCTGTGAGAGCAGCTTTGA |
| CTGF | CTGF -F | AAAAGTGCATCCGTACTCCCA |
|  | CTGF -R | CCGTCGGTACATACTCCACAG |
| CDX2 | CDX2-F | CCAATGACAACGCCTCCTG |
|  | CDX2-R | TGGTGCAGCCAGAAAGCTC |
| CYR61 | CYR61-F | AGCCTCGCATCCTATACAACC |
|  | CYR61-R | TTCTTTCACAAGGCGGCACTC |
| YAP | YAP-F | CCTGCGTAGCCAGTTACCAA |
|  | YAP-R | CCATCTCATCCACACTGTTC |
| USP7 | USP7-F | CGGTGTTGTGTCCATCACTC |
|  | USP7-R | AGTTGAGCGAGCCCGAG |
| β-actin | β-actin-F | ACTCTTCCAGCCTTCCTTCC |
|  | β-actin-R | CGTCATACTCCTGCTTGCTG |
