# Supplementary material for: Serine Metabolism Regulates YAP Activity Through USP7 in Colon Cancer
Source: Front Cell Dev Biol. 2021 May 12;9:639111. doi: 10.3389/fcell.2021.639111 (PMC8152669; doi:10.3389/fcell.2021.639111)
Supplement: Supplementary file 2 [file Table_2.DOCX]

**Supplemental Table 2.** Antibodies for immunoblotting analysis

| Antibody | Company | Cat No. |
| --- | --- | --- |
| PHGDH | proteintech | 14719-1-AP |
| PSAT1 | proteintech | 10501-1-AP |
| PSPH | proteintech | 14513-1-AP |
| PARP1 | proteintech | 13371-1-AP |
| YAP1 | proteintech | 13584-1-AP |
| Enolase-1 | CST | #3810 |
| Histone H3(D1H2) XP | CST | #4499 |
| α-Tubulin | CST | #2144 |
| Anti-Flag | CST | #2368 |
| Anti-HA | CST | #3724 |
| HAUSP/USP7 | CST | #4833 |
| β-Actin | CST | #3700 |
| Cyclin D1 | CST | #2922 |
| CDX2 | Abcam | ab76541 |
| SATB2 | Abcam | ab92446 |
| Ubiquitin | Abcam | ab134953 |
