# Supplementary material for: Serine Metabolism Regulates YAP Activity Through USP7 in Colon Cancer
Source: Front Cell Dev Biol. 2021 May 12;9:639111. doi: 10.3389/fcell.2021.639111 (PMC8152669; doi:10.3389/fcell.2021.639111)
Supplement: Supplementary file 4 [file Data_Sheet_1.docx]

**Supplementary Figure Legends**

**Figure S1. Effect of serine deficiency on cell apoptosis.**

(A) Apoptosis assay for SW620, SW480, LOVO and HCT116 cell lines cultured in basic and serine-deprived medium for 24 h were detected by flow cytometry.

**Figure S2. *De novo* serine synthesis pathway in colon cancer cells.**

(A) L-Serine synthesis pathway. On the one hand, PHGDH catalyzes the oxidation of 3-PG to 3-PHP and meanwhile the cofactor NAD^+^ was consumed into NADH. The subsequent transamination reaction is catalyzed by PSAT, which uses Glu as nitrogen donor and thereby produces 3-PS and α-KG into TCA cycle. Dephosphorylation of phosphoserine by PSPH gives rise to serine, then participating in one-carbon metabolism. On the other hand, serine can be obtained by transporters, like SLC7A6 and SLC1A4. PHGDH: 3-phosphoglycerate; 3-PG: 3-phosphoglycerate; 3-PHP: 3-phosphohydroxypyruvate; PSAT: phosphoserine aminotransferase; Glu: glutamate; 3-PS: 3-phosphoserine; α-KG: α-ketoglutarate; TCA: tricarboxylic acid; PSPH: phosphoserine phosphatase.

(B) qRT-PCR determined the expression of PHGDH, PSAT1 and PSPH mRNA in SW620 and SW620-S cells as indicated. Error bars represent mean ± SD (n = 3). * *p*<0.05.

(C) Immunoblotting analysis of PHGDH, PSAT1 and PSPH protein expression in SW620, SW620-S, LOVO and LOVO-S cell lines. The PHGDH, PSAT1 and PSPH levels were quantified against β-Actin.

(D) Discriminant analysis of orthogonal partial least square method (OPLS-DA) score plot of metabolites derived from SW620 and SW620-S cultured with RPMI media contain serine (SW620, 30mg/L) or no serine (SW620-S) with addition of 10% FBS for 24 h. R^2^X=0.891, R^2^Y=1, Q^2^=0.993. Blue circle corresponds to control group; green circle corresponds to model group.

(E) S-plot analysis of significantly changed metabolites in SW620 and SW620-S cells. Green circle corresponds to non-significance; red circle corresponds to significance.

(F) Node plot showing matched pathways according to significance (*p*-value) as determined by pathway enrichment analysis (y-axis), and pathways impact as determined by topology analysis (x-axis). Nodes in red indicate significance (-log10 (*p*) >1.3), and the size of the nodes indicate impact.

**Figure S3. Inhibition of d*e novo* serine synthesis pathway impedes the growth of colon cancer cells.**

(A) The cell proliferation of SW620-S treated with or without CBR5884 was determined by cell counting. Error bars represent mean ± SD (n = 3). * *p*<0.05.

(B) Discriminant analysis of orthogonal partial least square method (OPLS-DA) score plot of metabolites derived from SW620-S treated with or without 10μM CBR5884 for 24 h. R^2^X=1, R^2^Y=1, Q^2^=1. Blue circle corresponds to control group; yellow circle corresponds to model group.

(C) Heat map of significantly changed metabolites in SW620-S and SW620-S-CBR5884.

(D) S-plot analysis of significantly changed metabolites in SW620-S and SW620-S- CBR5884. Green circle corresponds to non-significance; red circle corresponds to significance.

(E) Node plot showing matched pathways according to significance (*p*-value) as determined by pathway enrichment analysis (y-axis), and pathways impact as determined by topology analysis (x-axis). Nodes in red indicate significance (-log_10_(*p*)>1.3), and the size of the nodes indicate impact.

**Figure S4. YAP target genes was activated in serine-starvation-resistant cells.**

(A) Immunoblotting analysis of Enolase 1 and α-tubulin protein expression in SW620, SW620-S, LOVO and LOVO-S cell lines.

(B) qRT-PCR analysis of CTGF, CDX2 and CYR61 mRNA expression in SW620 and SW620-S. Error bars represent mean ± SD (n = 3). * *p*<0.05.

**Figure S5. Serine deubiquitinates YAP by promoting the interaction between USP7 and YAP.**

(A) qRT-PCR analysis of CTGF mRNA expression in SW620 treated with serine deficiency medium for 1 day, 2 days and 4 days. Error bars represent mean ± SD (n = 3). * *p*<0.05.

(B) SW620 and LOVO were cultured with medium without serine and harvested at the indicated days, protein levels of PHGDH were analyzed by immunoblotting.

(C) Quantification of YAP protein levels for each point in SW620 and LOVO cells treated with different concentrations of serine was determined by densitometry. The concentrations of 1×Ser, 10×Ser and 20×Ser are 30 μg/mL, 300 μg/mL and 600 μg/mL, respectively. * *p*<0.05.

(D) mRNA levels of CTGF and CDX2 in SW620 treated with different concentrations of serine were detected by qRT-PCR. The concentrations of 1×Ser, 4×Ser, 10×Ser and 16×Ser are 30 μg/mL, 120 μg/mL, 300 μg/mL and 480 μg/mL, respectively. Error bars represent mean ± SD (n = 3). * *p*<0.05.

(E) mRNA levels of YAP in SW620 treated with different concentrations of serine were detected by qRT-PCR. The concentrations of 1×Ser, 4×Ser, 10×Ser and 16×Ser are 30 μg/mL, 120 μg/mL, 300 μg/mL and 480 μg/mL, respectively. Error bars represent mean ± SD (n = 3). * *p*<0.05.

(F) Quantification of YAP protein levels for each point in SW620 cells with different treatment was determined by densitometry. * *p*<0.05.

(G) YAP protein expression in SW620-S treated with different concentrations of PR619 or Degrasyn was detected by Immunoblotting.

(H) SW620-S transfected with indicated siRNA, followed by qRT-PCR analysis being performed with USP7, CTGF and CDX2 primers.

**Figure S6. YAP reversely regulates USP7 and serine metabolism.**

(A and B) Heatmap and volcano plot displaying global significantly changed genes in SW620-S-VP cells compared with SW620-S-Con cells. (B) Each dot represents a gene, and the red dots represent up-regulated genes (1711), while the blue dots represent down-regulated genes (2607) (adjust *p* < 0.05) in SW620-S-VP.

(C) KEGG analysis of signaling pathways involving in differentially expressed genes in SW620-S-VP cells compared with SW620-S-Con cells. Each bar graph represents a pathway, and the length of the bar shows differential gene number in that pathway.

(D) List of associated deubiquitination enzymes significant differentially expressed between SW620-S-VP and SW620-S-Con cells.

(E) Discriminant analysis of orthogonal partial least square method (OPLS-DA) score plot of metabolites derived from SW620-S treated with or without 5 μM Verteporfin (VP) for 24 h. R^2^X=0.78, R^2^Y=0.999, Q^2^=0.984. Blue circle corresponds to control group; red circle corresponds to model group.

(F) Heat map of significantly changed metabolites in SW620-S-Con and SW620-S-VP.

(G) S-plot analysis of significantly changed metabolites in SW620-S-Con and SW620-S-VP. Green circle corresponds to non-significance; red circle corresponds to significance.

(H) Node plot showing matched pathways according to significance (*p*-value) as determined by pathway enrichment analysis (y-axis), and pathways impact as determined by topology analysis (x-axis). Nodes in red indicate significance (-log_10_(*p*)>1.3), and the size of the nodes indicate impact.

**Figure S7. Expression of YAP in colon cancer tissues.**

(A) YAP1 expression levels in 41 paired colon cancer and non-tumors tissue, based on TCGA RNA-sequencing data, *p* = 0.0003489.

(B) The gene expression level of YAP negatively correlates with the OS (overall survival) of patients with colon cancer (Kaplan Meier curve was calculated with cutoff value according to the median of expression of YAP), *p* = 0.035

(C) YAP1 expression levels in different stages of colon cancer, based on TCGA RNA-sequencing data, *p* = 0.0937.

(D) Samples were obtained from patients with colon cancer. Immunoblotting analysis of YAP protein expression in paired samples of para-cancerous region (N) and tumor region (T) from the same patient.

(E) Quantification of YAP protein level for each patient was determined by densitometry. Error bars represent mean ± SD (n = 3). * *p*<0.05.

**Figure S8. Effects of YAP inhibitors on organoid morphology of colon cancer.**

(A) Changes in the number and morphological structure of colon carcinoma organoids after treatment with Verteporfin (VP), one of YAP inhibitors, under different concentrations (0, 200,1000, 5000 and 25000 nM). Scale bars: 100 μm.

(B) Oncomine data mining analysis of YAP mRNA in colon carcinomas with and without metastases. M0: colon carcinomas without metastases (20 patients); M1: colon carcinomas with metastases (30 patients).
