# Supplementary figures and images for: Serine Metabolism Regulates YAP Activity Through USP7 in Colon Cancer
Source: Front Cell Dev Biol. 2021 May 12;9:639111. doi: 10.3389/fcell.2021.639111 (PMC8152669; doi:10.3389/fcell.2021.639111)

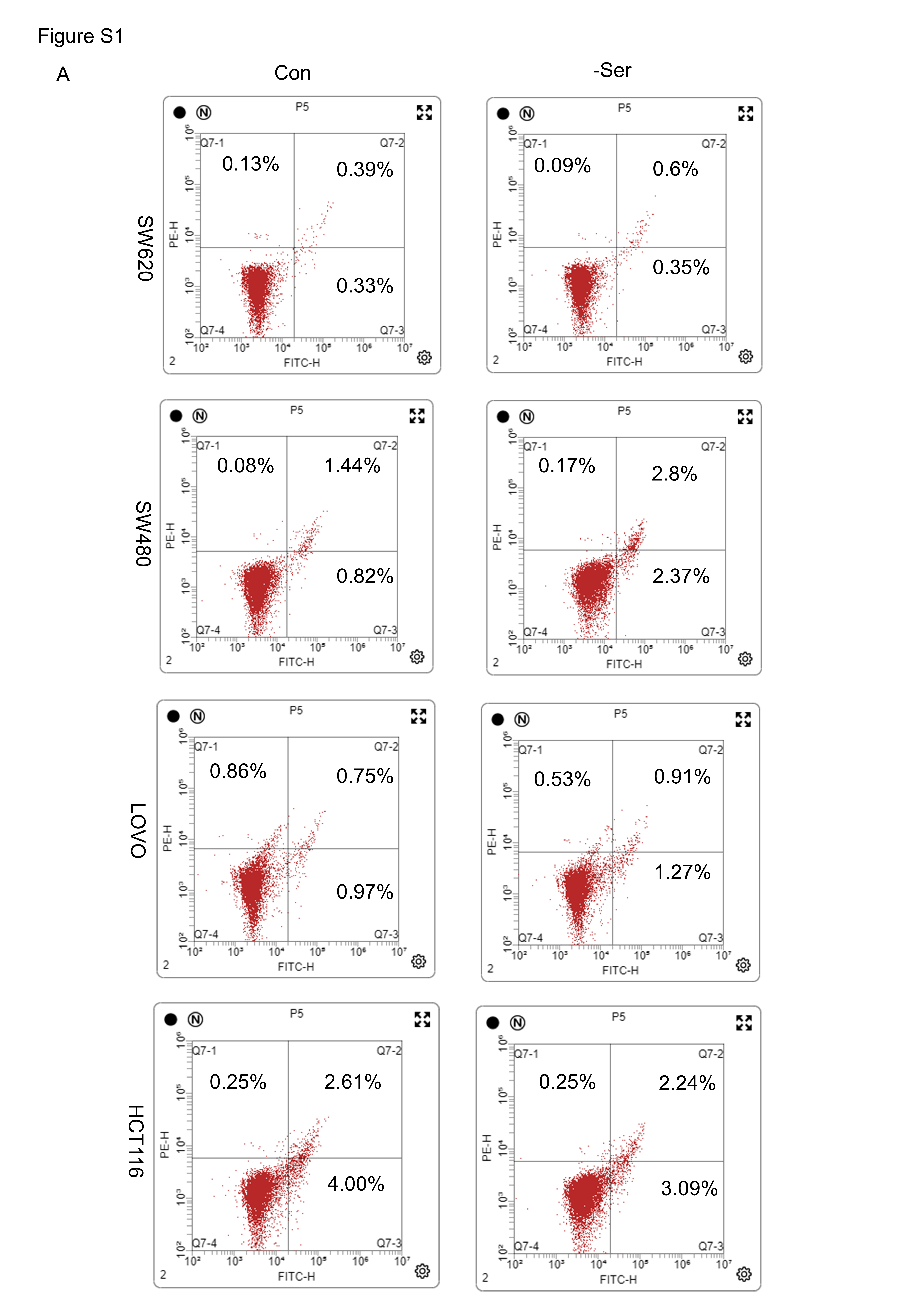

Supplement: Supplementary file 5 [file Image_1.TIF]

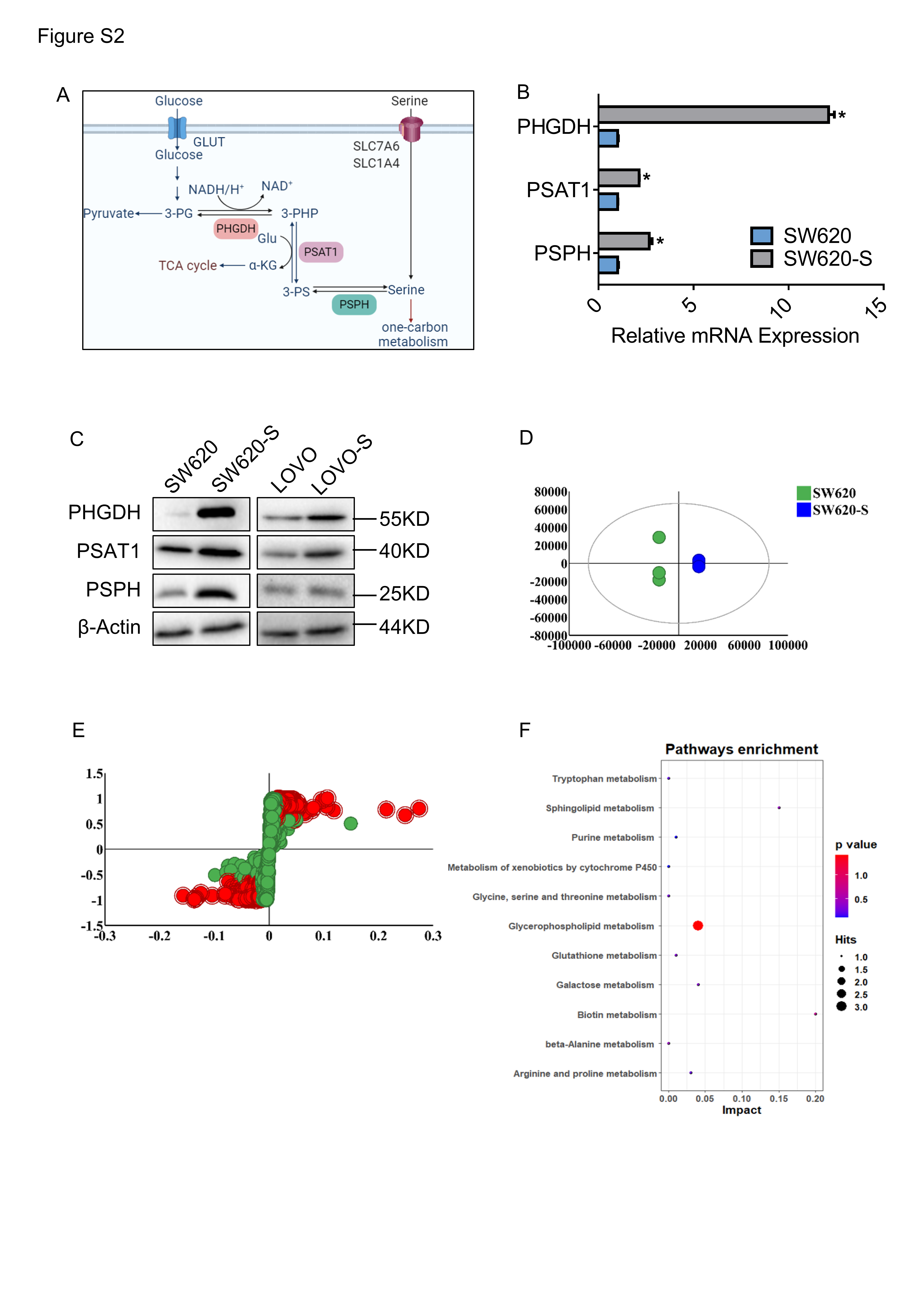

Supplement: Supplementary file 6 [file Image_2.TIF]

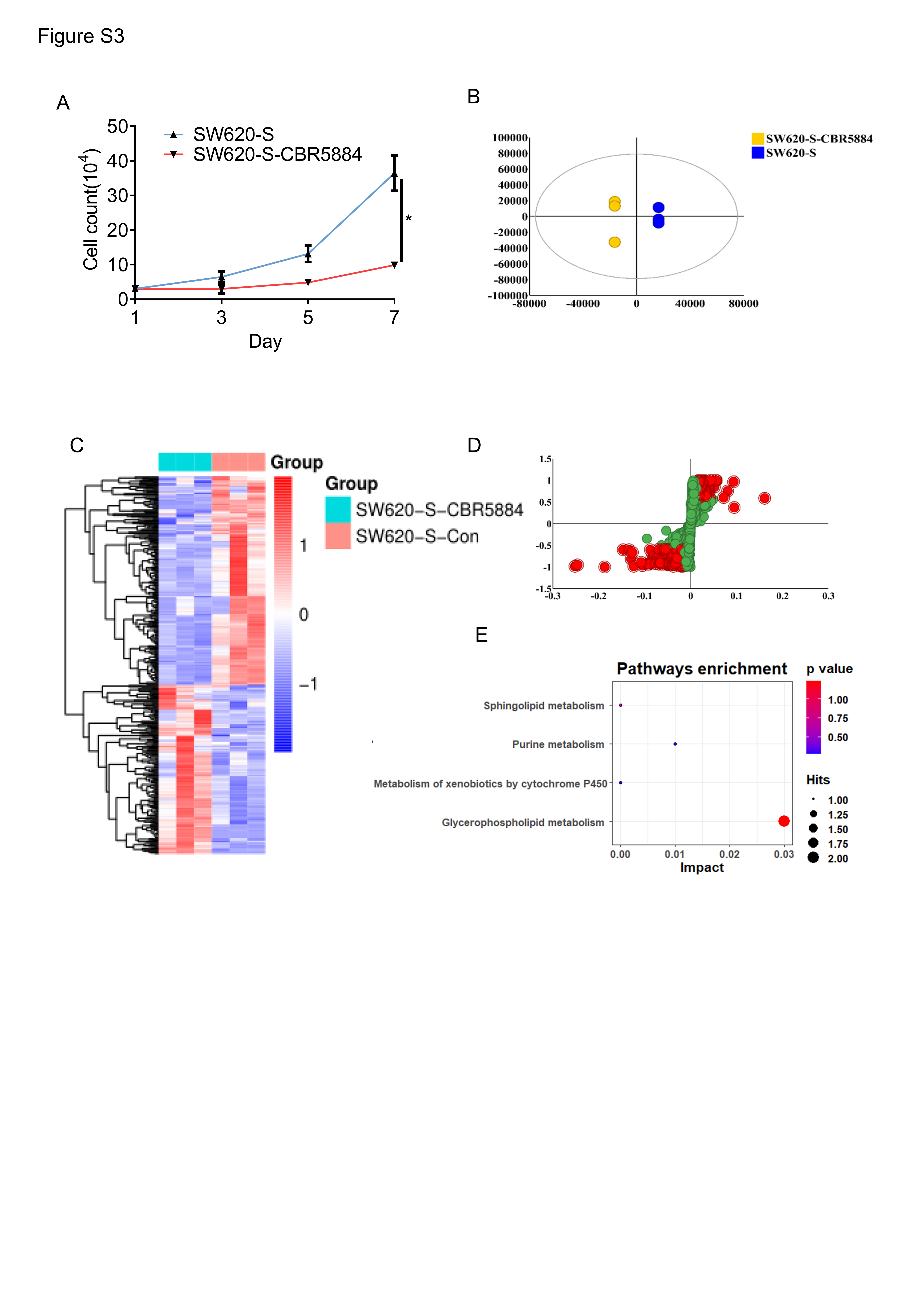

Supplement: Supplementary file 7 [file Image_3.TIF]

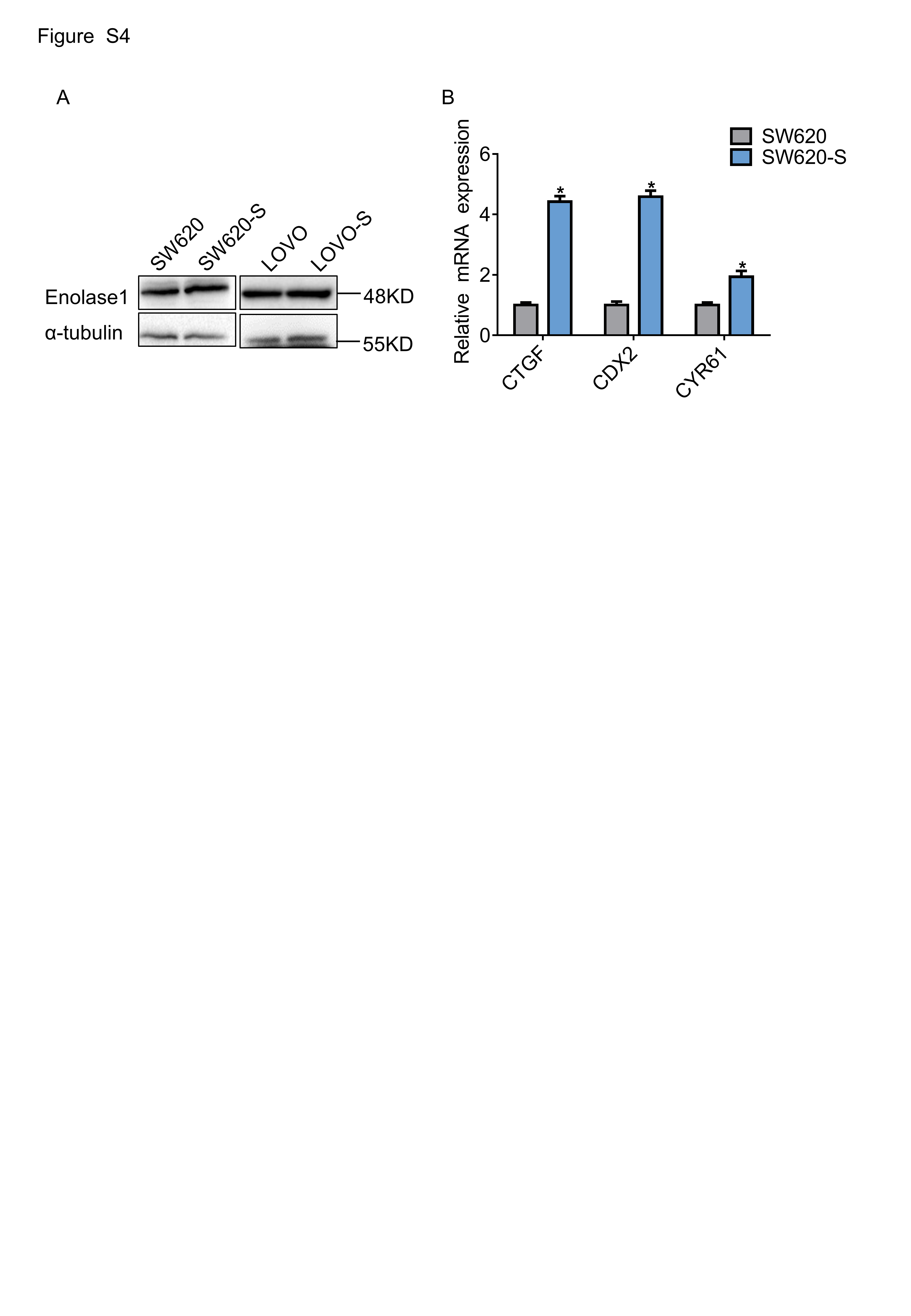

Supplement: Supplementary file 8 [file Image_4.TIF]

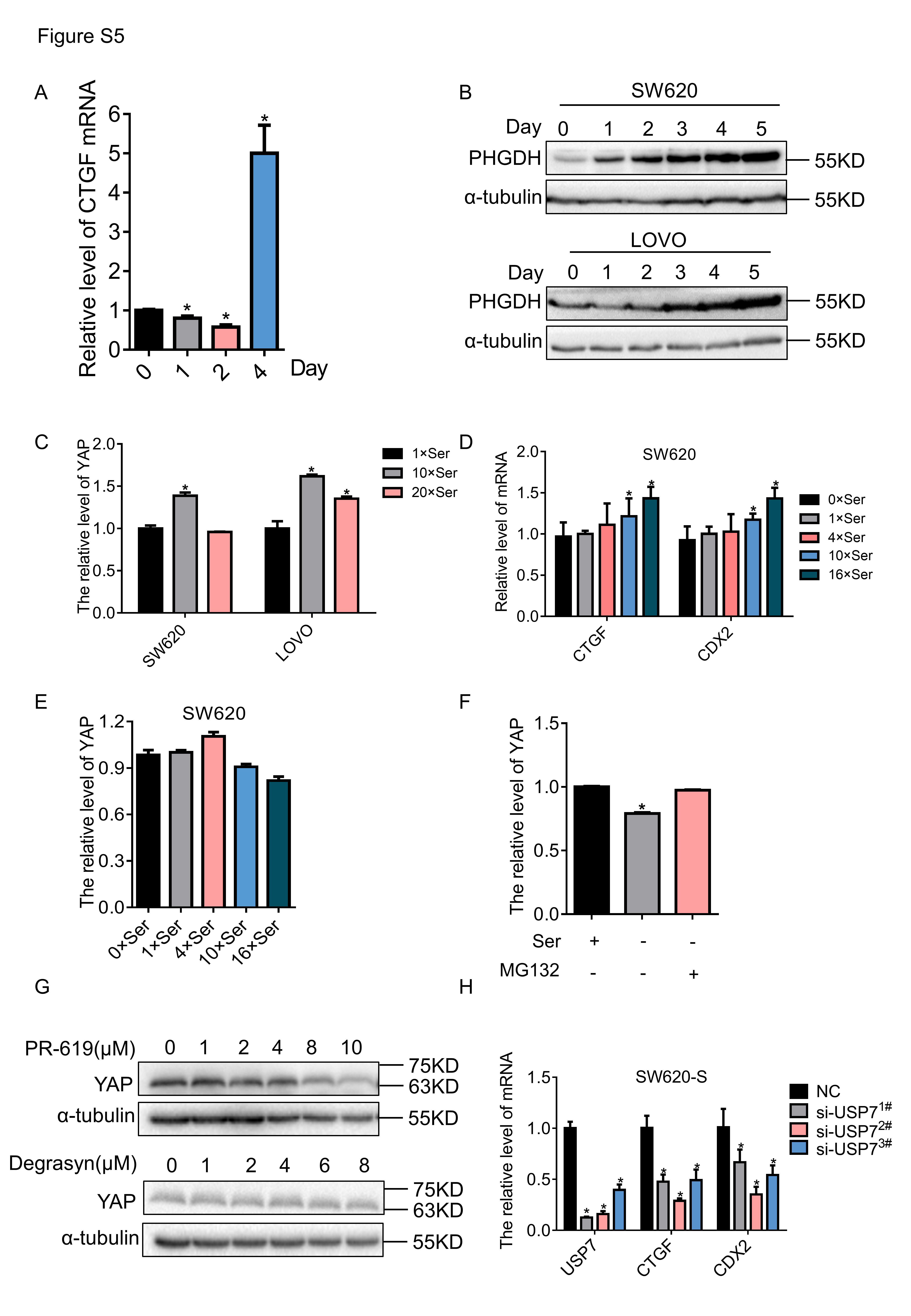

Supplement: Supplementary file 9 [file Image_5.TIF]

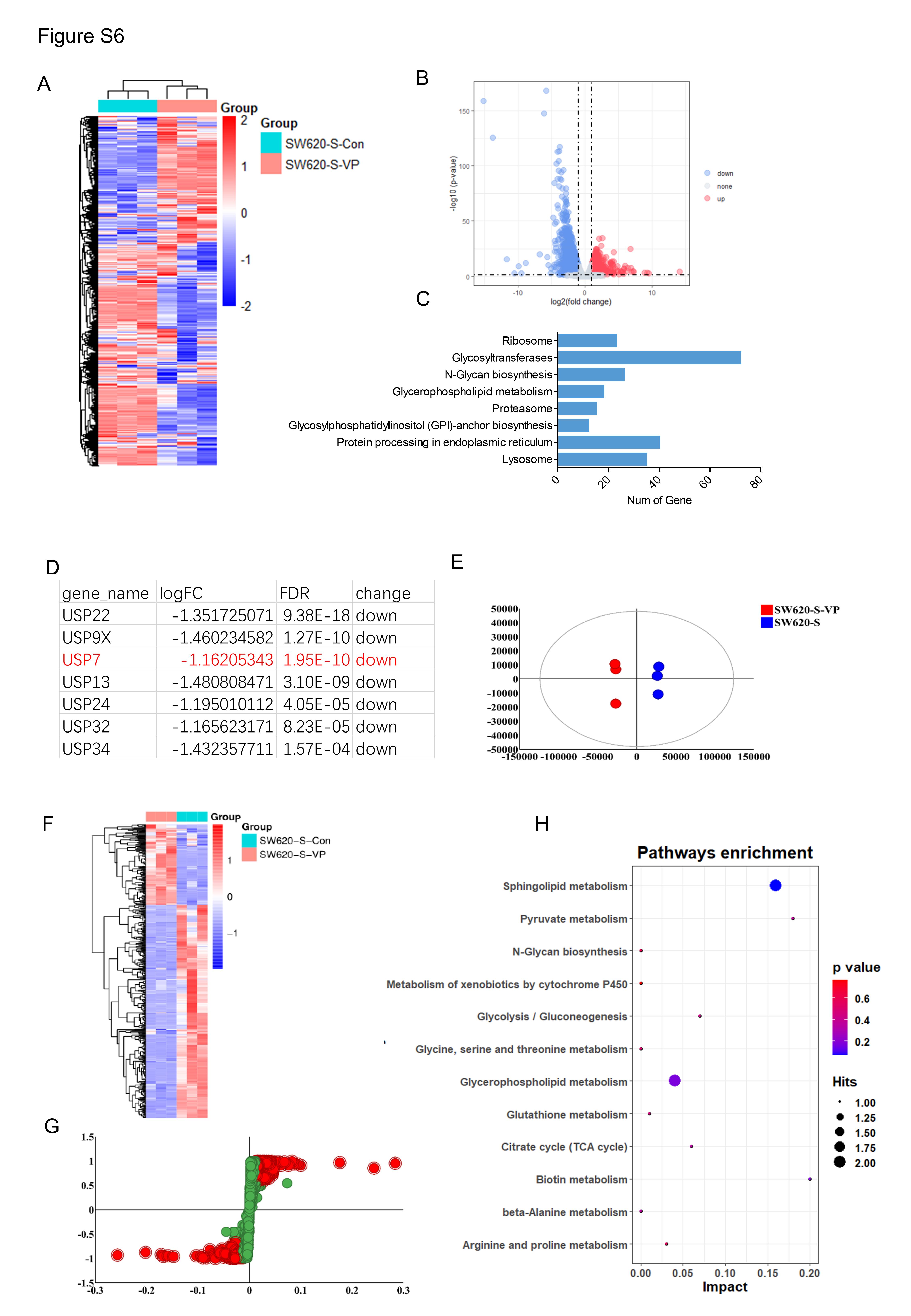

Supplement: Supplementary file 10 [file Image_6.TIF]

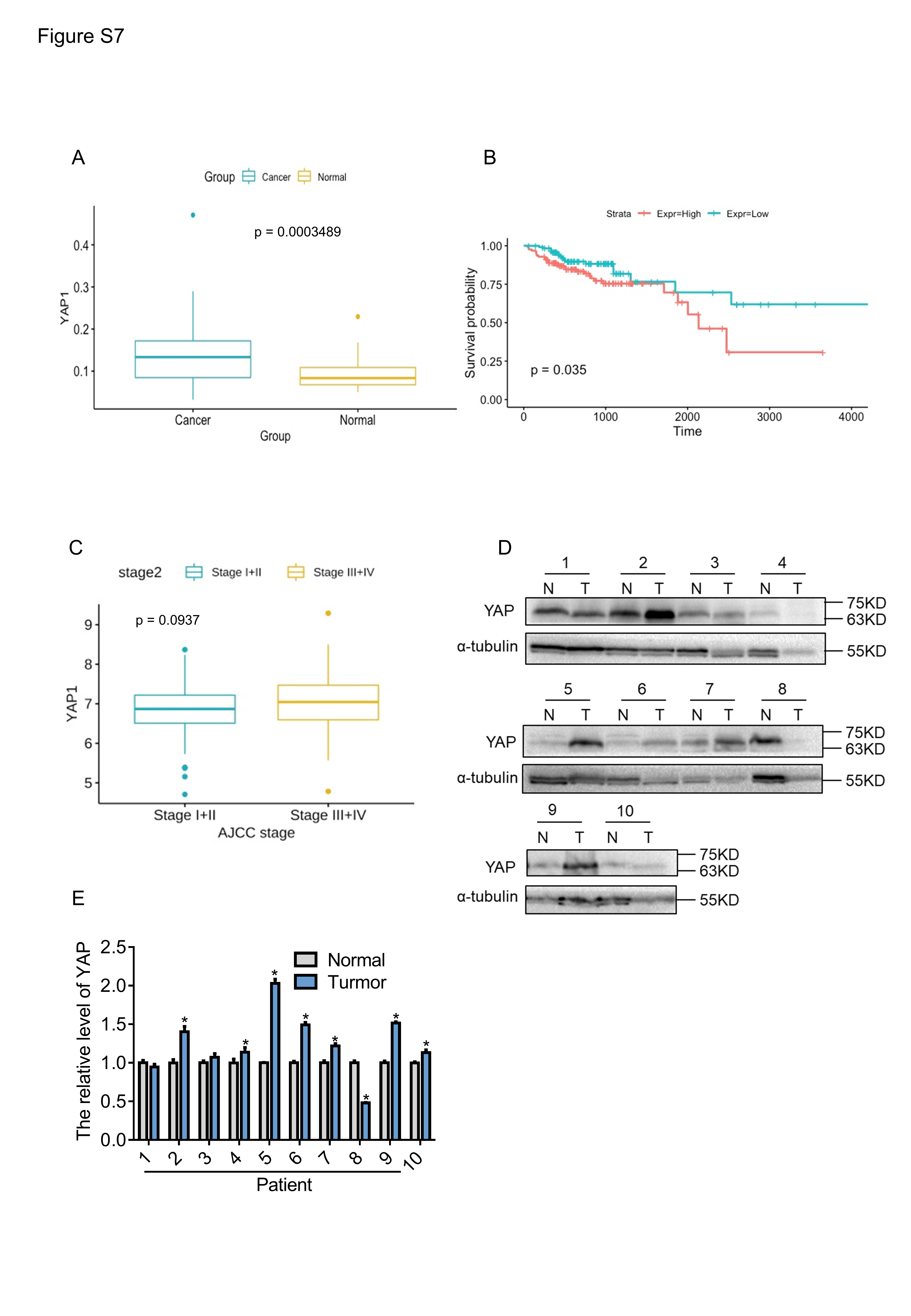

Supplement: Supplementary file 11 [file Image_7.TIF]

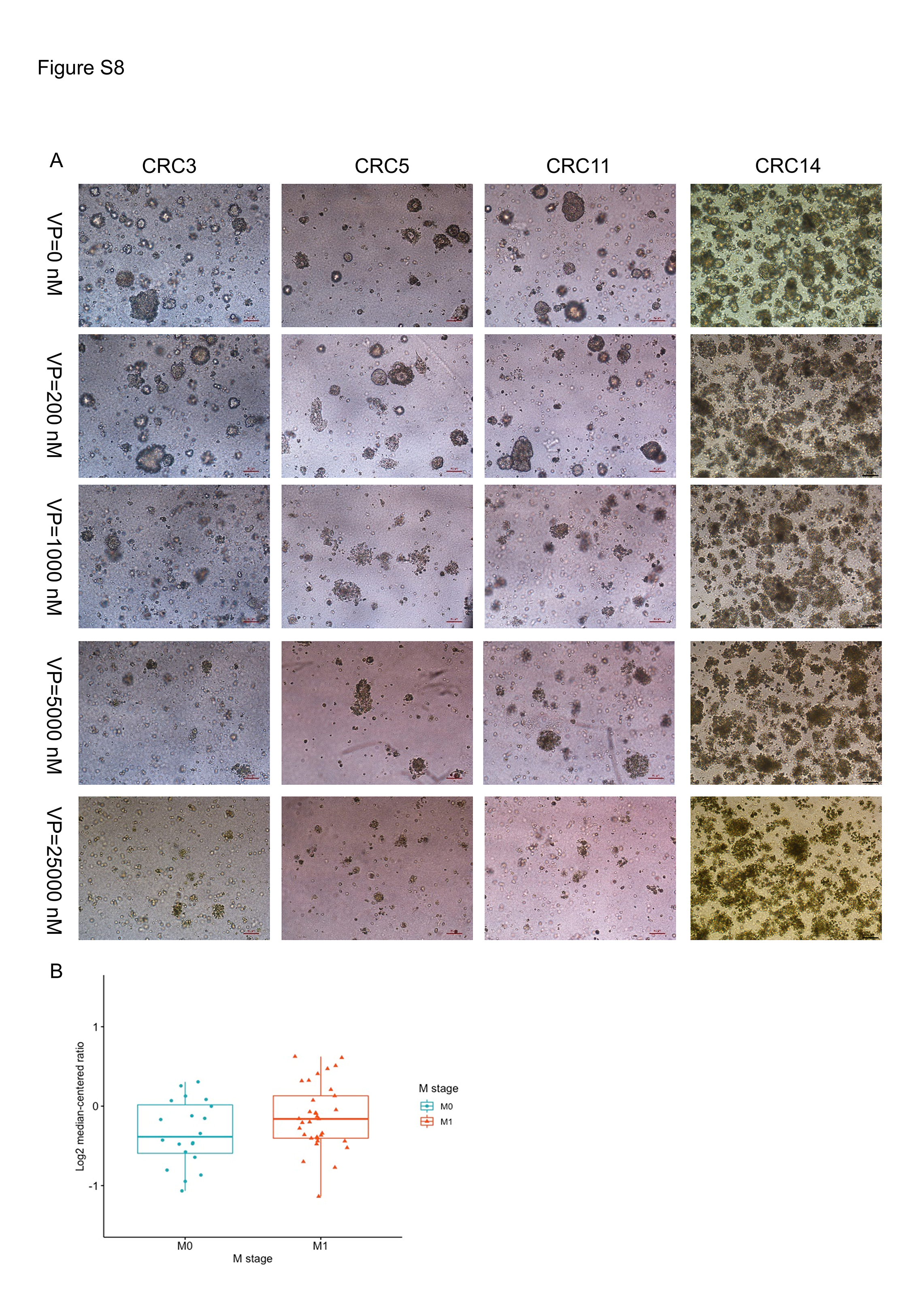

Supplement: Supplementary file 12 [file Image_8.TIF]
